# Respiratory Distress Secondary to Esophageal Foreign Body. A Case Report

**DOI:** 10.1100/tsw.2006.08

**Published:** 2006-01-17

**Authors:** Jacob Urkin, Yair Bar-David

**Affiliations:** Primary Care Unit, Division of Health in the Community, Faculty of Health Sciences, Ben-Gurion University of the Negev and Clalit Health Service, Beer-Sheva, Israel

**Keywords:** child health, pediatrics, esophageal foreign body, ingestion, childhood accidents, prevention, Israel

## Abstract

The ingestion or aspiration of a foreign body is a common, but preventable occurrence in childhood. Primary healthcare personnel should alert parents to the risk of swallowing a foreign object, the signs and the need for immediate medical attention. It should be emphasized that protecting children from access to objects that can be swallowed or aspirated is the best preventive measure. A case of an eight year old child, who had swallowed a marble ball is presented and the symptoms and intervention discussed. Medical staff should be aware of the symptomatic variation in ingested foreign body presentation and the importance of rapid diagnosis and management.

## INTRODUCTION

Esophageal foreign body (EFB) is a common problem that has the potential to cause significant local damage or to compromise breathing[1]. Primary care physicians should be aware of the variability of EFB presentation and the pitfalls in its diagnosis and management.

## CASE DESCRIPTION

An 8-year-old boy presented at the pediatric emergency room with cough and drooling. The resident attempted to perform the Heimlich procedure (http://www.heimlichinstitute.org/howtodo.html) in order to dislodge a suspected pharyngeal foreign body. Brief history revealed that the boy had swallowed a marble ball and since then had been coughing continuously. A quick physical examination revealed that the boy was alert with mild respiratory difficulty. His breathing rate was 28 per minute, without any retractions. Color was normal, with no signs of cyanosis. He was drooling. There was no stridor and the boy was able to talk, however, talking was interrupted by repeated episodes of intense coughing. Oxygen saturation was 97%, blood pressure 100/66, pulse 100, temperature 36.8°C. Based on the history and physical findings, it seemed that the foreign body was lodged in the upper esophagus and breathing was not critically compromised. On chest and neck radiogram, a spherical ball was noted just above the thoracic inlet, pushing the posterior wall of the trachea anteriorly, thus narrowing the lumen (see Fig. 1). The ENT (ear, nose, and throat) consultant was alerted and the child admitted for bronchoscopy and observation.

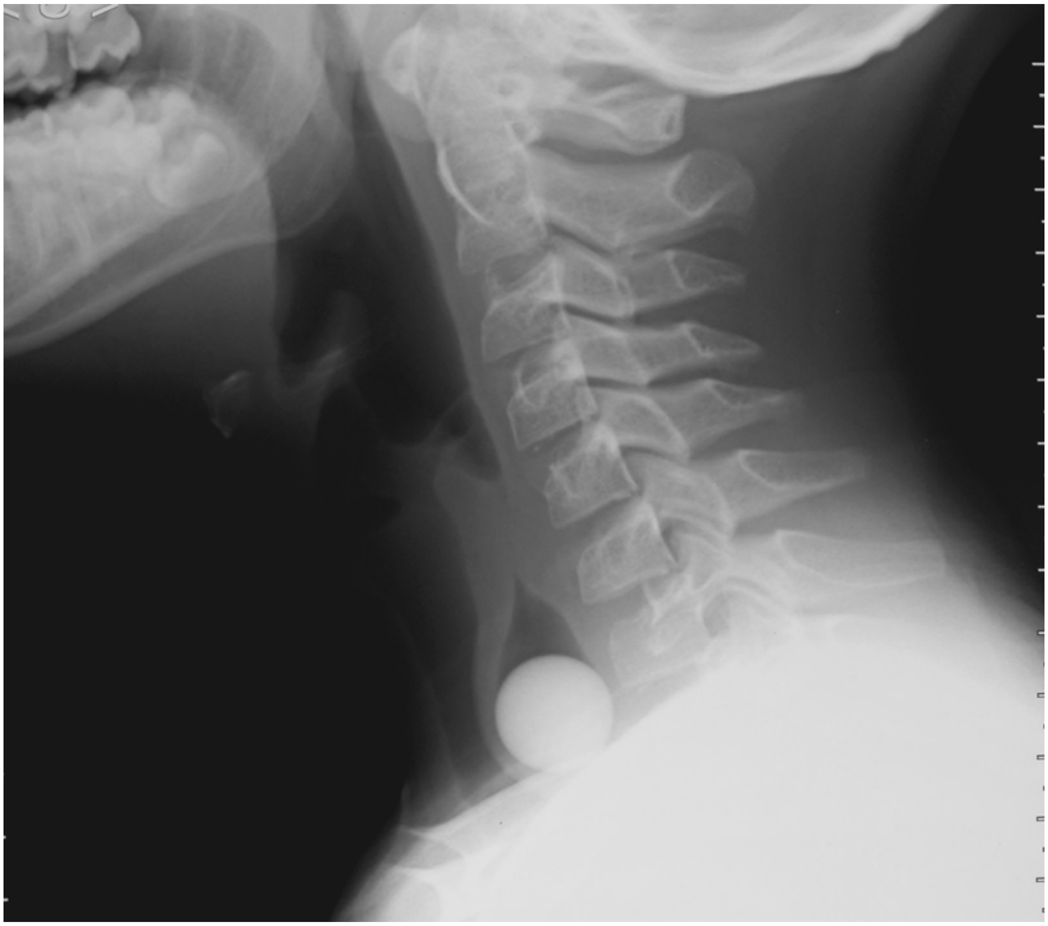

**FIGURE 1.** Lateral radiogram of the neck with a spherical ball above the thoracic inlet, pushing the posterior wall of the trachea anteriorly.

IV-fluids, papaverin, and analgesics were initiated. On a second radiograph, 5 h later, the ball was in the same location. Using a rigid bronchoscope, the ball was observed in mid-esophagus. An unsuccessful attempt was made to pull the ball out and therefore instead pushed into the stomach. The rest of the hospitalization was uneventful and the boy was discharged the next day. On follow-up conversation with the parents, no sequelae were reported.

## DISCUSSION

Respiratory symptoms are not necessarily a sign of a primary problem in the respiratory system. Esophageal foreign body (EFB) is a possibility that should be considered[1].

Although most commonly described EFBs are coins, other ingested objects include fish and chicken bones, toy parts, jewels, batteries, needles, pins, balls, and buttons[1,2,3]. As some children show no symptoms, a high level of suspicion is needed in cases where ingestion of a foreign body was not witnessed. In some cases, history alone is enough to justify further evaluation[3]. The most common method to demonstrate EFB is by performing neck and chest films. Anterior, posterior, and lateral films are needed in order not to miss objects that have one narrow plane. Radio-opaque objects may present as they push on other structures or as a result of complications.

Bones and sharp objects have a tendency to get stuck at the level of the oropharynx or upper esophagus. In these cases, direct vision or laryngoscopic-aided view could be enough to make the diagnosis and to extract the foreign body[2]. Other EFBs that do not pass to the stomach tend to get impacted at one of the three following levels: the thoracic outlet (70%), mid-esophagus, where the aortic arch and the carina overlap (15%), and at the lower esophageal sphincter (15%)[1]. The presentations of EFB include gastroesophageal and respiratory signs and symptoms, as well as general ones. Few children need emergency airway management.

The symptoms of the patient presented were interpreted initially as oropharyngeal obstruction. However, spontaneous breathing and the child's ability to talk negated the need for abdominal thrust. Our patient had swallowed a marble ball that could easily be seen on the X-ray film. The ball in the esophagus was obviously pushing the posterior elastic wall toward the anterior wall of the trachea and causing narrowing of the tracheal lumen. Potentially this may lead to breathing difficulties similar to those with orolaryngeal, laryngeal, or tracheal foreign body. As long as there is effective breathing, it is not recommended to dislodge EFB by abdominal thrust, nor is it recommended to induce vomiting or intubate the airway.

When there is no breathing difficulty, a case of acute ingestion of foreign body can be observed for a couple of hours and checked as to whether the EFB has passed to the stomach. The exceptions to this are sharp or potentially corrosive objects that should be removed as soon as possible[4]. In the described patient, the ball was at the same esophageal level 5 h after admission and extraction of the ball was attempted. The most common method of EFB extraction is by endoscopy[2,5].

Surgical removal of EFB is rarely needed. This method is indicated in cases of local complications due to the foreign body itself or to the extracting procedure. Delay in the diagnosis of EFB or failure to diagnose more than one foreign body can be associated with severe complications, which include mediastinitis, failure to thrive, pneumothorax, abscess, and stricture[6,7].

## CONCLUSION

The ingestion or aspiration of a foreign body is a common, but preventable, childhood occurrence. Primary healthcare personnel should alert parents to the risk of swallowing a foreign object, including the signs of EFB and the need for immediate medical attention. It should be emphasized that protecting children from access to objects that can be swallowed or aspirated is the best method of preventing EFB[7]. Medical staff should be aware of the variation in EFB presentation and of the importance of its rapid diagnosis and management.

## BIOSKETCHES

**Jacob Urkin MD, MPH**, is a lecturer in pediatrics and director of the Primary Pediatric Unit and director of the Maternal and Child program of the MPH program at the Faculty of Health Sciences, Ben Gurion University of the Negev, Beer-Sheva, Israel. Major academic interests are primary care, community pediatrics, well baby care services, school health, and medical education. E-mail: jacobur@clalit.org.il

**Yair Bar-David, MD, MHA,** is a lecturer in pediatrics affiliated with the Primary Pediatric Unit, Faculty of Health Sciences, Ben Gurion University of the Negev and the Zusman Child Development Center, Division of Pediatrics, Soroka University Medical Center, Beer-Sheva, Israel. E-mail: medcad@bgumail.bgu.ac.il
